# Food Industry Byproducts as Starting Material for Innovative, Green Feed Formulation: A Sustainable Alternative for Poultry Feeding

**DOI:** 10.3390/molecules27154735

**Published:** 2022-07-25

**Authors:** Leonardo Brunetti, Rosalba Leuci, Maria Antonietta Colonna, Rossana Carrieri, Francesco Emanuele Celentano, Giancarlo Bozzo, Fulvio Loiodice, Maria Selvaggi, Vincenzo Tufarelli, Luca Piemontese

**Affiliations:** 1 Department of Pharmacy—Pharmaceutical Sciences, University of Bari Aldo Moro, Via E. Orabona 4, 70125 Bari, Italy; leonardo.brunetti@uniba.it (L.B.); rosalba.leuci@uniba.it (R.L.); r.carrieri9@studenti.uniba.it (R.C.); fulvio.loiodice@uniba.it (F.L.); 2 Department of Agricultural and Environmental Science (DISAAT), University of Bari Aldo Moro, Via E. Orabona 4, 70125 Bari, Italy; mariaantonietta.colonna@uniba.it (M.A.C.); maria.selvaggi@uniba.it (M.S.); 3 Department of Law, University of Bari Aldo Moro, Corso Italia 23, 70121 Bari, Italy; francesco.celentano@uniba.it; 4 Department of Veterinary Medicine, University of Bari Aldo Moro, Strada Provinciale per Casamassima, km 3, 70010 Valenzano, Italy; giancarlo.bozzo@uniba.it; 5 Department of DETO, Section of Veterinary Science and Animal Production, University of Study of Bari “Aldo Moro”, Strada Provinciale per Casamassima, km 3, 70010 Valenzano, Italy; vincenzo.tufarelli@uniba.it

**Keywords:** poultry, grape pomace, artichoke, byproducts, Deep Eutectic Solvents (DESs), feed, supplements, antioxidants, eco-friendly, extraction

## Abstract

Rising global populations and enhanced standards of living in so-called developing countries have led to an increased demand of food, in particular meat, worldwide. While increasing the production of broiler meat could be a potential solution to this problem, broiler meat is plagued by health concerns, such as the development of antimicrobial resistance and lower meat quality. For this reason, the supplementation of poultry feed with vitamins and antioxidant compounds, such as polyphenols, has become an attractive prospect for research in this sector. Such supplements could be obtained by extraction of agricultural byproducts (in particular, grape pomaces and artichoke leaves and bracts), thus contributing to reductions in the total amount of waste biomass produced by the agricultural industry. In this review, the effects of poultry feed supplementation with bioactive extracts from grape pomace (skins and/or seeds), as well as extracts from artichoke leaves and bracts, were explored. Moreover, the various methods that have been employed to obtain extracts from these and other agricultural byproducts were listed and described, with a particular focus on novel, eco-friendly extraction methods (using, for example, innovative and biocompatible solvents like Deep Eutectic Solvents (DESs)) that could reduce the costs and energy consumption of these procedures, with similar or higher yields compared to standard methods.

## 1. Introduction

The continuous increase in the world’s population (predicted to reach nine billion by 2050), combined with scarcity of natural resources such as water and arable soil, is a looming threat to worldwide food availability and safety. According to the most recent FAO estimates, between 720 and 811 million people faced hunger in 2020, while 2.37 billion people did not have adequate food in the same year. Both of these numbers were substantially higher than those reported in 2019. Of course, the high cost of maintaining a healthy diet, combined with persistent poverty and income inequality, was intensified by the effects of the COVID-19 pandemic [1], as well as by international tensions in eastern Europe in 2022.

In 1996, at the World Food Summit, Heads of State and Government reaffirmed “the right of everyone to have access to safe and nutritious food, consistent with the right to adequate food and the fundamental right of everyone to be free from hunger” [2]. This fundamental right is essential to guarantee many other human rights recognized at both the international and national level, namely the rights to health, privacy and family life, and a healthy environment. This last, and more recent, right, is strictly related to the production of food, since, in recent decades, science and international fora—such as the United Nations and its Agencies—have clarified that livestock and agriculture are at the top of the list of climate change causes [3].

The world’s population increase, along with rising incomes in developing countries, has driven up global food demand, which is expected to increase by 35–56% more between 2010 and 2050 [4]. This will shape agricultural markets in ways never before seen. As a consequence, farmers will need to increase crop production, either by increasing the amount of agricultural land on which crops are grown, or by enhancing productivity on existing agricultural lands through fertilization and irrigation and adopting new methods like precision farming [5].

Due to this global emergency, the need for increased production of meat is easily understandable. In this context, poultry may make a substantial contribution to food security and nutrition, since broiler meat provides energy, protein, and essential micro-nutrients to humans. Among producing livestock species, poultry needs short production cycles, through which a wide range of agri-food byproducts and wastes can be used as feed and converted into edible meat and eggs. Therefore, the poultry sector is particularly attractive to small holders, both in rural and urban areas, and it could be a key asset for poverty alleviation, providing income and market participation [6].

While the productivity of broiler chicken is now higher than ever, thanks to genetic selection, improved animal nutrition, management, and dietary supplementation with antibiotics [7], poultry represents a threat to human health, especially as a vector of infectious diseases and because of its role in the development of antimicrobial resistance [8].

Thus, the sustainable productivity of the meat industry hinges on the discovery and development of novel dietary supplements that could mimic antibiotic growth promoters, among which prebiotics, probiotics, synbiotics, organic acids, essential oils, antibodies, enzymes and phytogenic feed additives can be enumerated [8]. The addition of these natural extracts can be useful for the growth performance of broilers since it does not negatively affect animal health or the quality of animal products for human consumption [9]. Moreover, antioxidant phenols and vitamins contained in plants exert beneficial effects on the reproductive health, immune system and growth of poultry by reducing the detrimental effects of free radicals and toxic metabolites [10,11].

Each year a large quantity of agro-industrial byproducts is generated as a result of the processing of agricultural crops, representing an environmental concern. Many of these agro-industrial residues have been used in animal diets, representing a major opportunity for the development of a circular economy, improving economic and environmental sustainability [12].

Circular production models aim to develop more efficient systems able to reduce both the consumption of natural resources and the production of wastes. Although biomass wastes generated along the food chain are not suitable for human consumption, they have important nutritional characteristics [13], thus representing valuable coproducts that are usable in animal nutrition [14,15].

Many coproducts obtained from fruit and vegetable processing have great potential in animal nutrition due to their considerable bioactive components (i.e., polyphenols, flavonoids and tannins) [12]. The inclusion of all the above-mentioned coproducts in animal diets could improve animal health and provide added value to animal products [16]. In fact, many reports have documented the extraction of antimicrobial and antioxidant products from agro-industrial byproducts such as rice bran, wheat germ, rice husk, vegetable peels, fruit seeds and essential oils from the peel or seeds of various plants [12].

Indeed, in addition to traditional extraction processes, much research has focused on novel extraction methods of these bioactive compounds from vegetable matrices, such as pressurized liquid extraction (PLE), microwave-assisted extraction (MAE) or ultrasound-assisted extraction (UAE), which are also less energy-intensive [17].

Extractions can be carried out using a variety of solvents, but if the end goal is to achieve a cycle of sustainable reuse of food waste, it is imperative that such methods follow the practices of green chemistry; namely, the use of green solvents [18,19]. Several liquids have been recognized as green solvents over the course of the past decades, including, but not limited to, bioethanol, compressed fluids (e.g., supercritical CO_2_, pressurized water) and Deep Eutectic Solvents (DESs) [19].

DESs were first described in 2003 and have gained increasing prominence ever since [20]. These solvents are composed of two or more nontoxic components, with one of them acting as Hydrogen Bond Acceptor (HBA) and the other as Hydrogen Bond Donor (HBD) [20,21]. The two species interact through hydrogen bonds, forming a eutectic mixture whose melting point is lower compared to that of each component [22]. Natural Deep Eutectic Solvents (NADESs) are a subclass of solvents made by natural molecules present in living cells, such as amino acids like HBA and organic acids or sugars like HBD [23]. As reported in recent papers, these media are used for the green extraction of various bioactive compounds from natural sources, for example: phenolic acids, flavonoids, polyphenols, saponins, carbohydrates and polyunsaturated fatty acids. This is largely thanks to properties such as low toxicity, noninflammability, high thermal stability and biodegradability, in addition to economic feasibility [19,22,23,24].

DESs are also suitable for the development of green extraction methods in the determination of food contaminants, such as ochratoxin A (OTA) in wheat [25] or aflatoxins in crops [26] and rice [27].

Choline chloride (ChCl), a natural quaternary ammonium salt, is the most used HBA, combined with several HBDs, such as urea, glycerol (Gly), and lactic acid (La). Most Ch-DESs have the advantage of preserving the bioactivity of extracted compounds. Moreover, the physicochemical properties (viscosity, pH, and polarity) can be adjusted by changing hydrogen bond donors [28,29]. ChCl is a source of choline, used as supplement in the broiler diet [30]. In poultry, the methyl group of choline is available due to the conversion of choline to betaine in the liver. Betaine hydrochloride can be also used as-is as an HBA for NADESs formulation [31]. Choline plays metabolic roles as constituent of the most important phospholipids, phosphatidylcholines, and as precursor of the neurotransmitter acetylcholine. It also plays an important role in preserving the integrity of some organs—for example, preventing hepatic steatosis [30].

Glycerol is a polyol used as a solvent, especially in the food and pharmaceutical industries. It is another typical component of DESs. It plays the role of HBD with different ammonium and phosphonium salts [32]. Like most DES components, glycerol is nontoxic, nonflammable and nonvolatile, and while its density and high boiling point make it hard to remove it by evaporation, its high biocompatibility does not require its removal before being included as ingredient in food and feed additives [19]. Indeed, glycerol is part of all triglycerides found in animal and plant tissues and, after absorption, it is converted into glucose or oxidated to produce energy via gluconeogenesis, glycolysis and the citric acid cycle, respectively [33]. Glycerol has shown significant capacity for the extraction of polyphenols in aqueous solutions from a variety of plant sources, such as olive leaves, apple, potato, eggplant and grapefruit peels—and, of course, grape pomace. Hydroglycerolic solvents afford higher extraction yields and faster extraction kinetics than hydroalcoholic mixtures. Used as a pure solvent, glycerol also performs better than ethanol and water in the extraction of polyphenols from mangosteen (*Garcinia mangostana*). Similar advantages were reported for hydroglycerolic extracts of medicinal and aromatic plants. At the same time, the use of glycerol-based DESs has been reported in the literature for similar extraction procedures from waste products, such as onion solid wastes and olive leaves, and from aromatic plants like pink savory (*Satureja thymbra*) and oregano (*Origanum dictamnus*) [19].

Other typical components of DESs include organic acids such as malic (Ma), tartaric (Ta), citric (Cit), oxalic (Oa) and ascorbic (Aa) acids, sugars such as fructose (Fru) and sorbose (Sor) and aminoacids such as proline (Pro).

**Figure 1:**
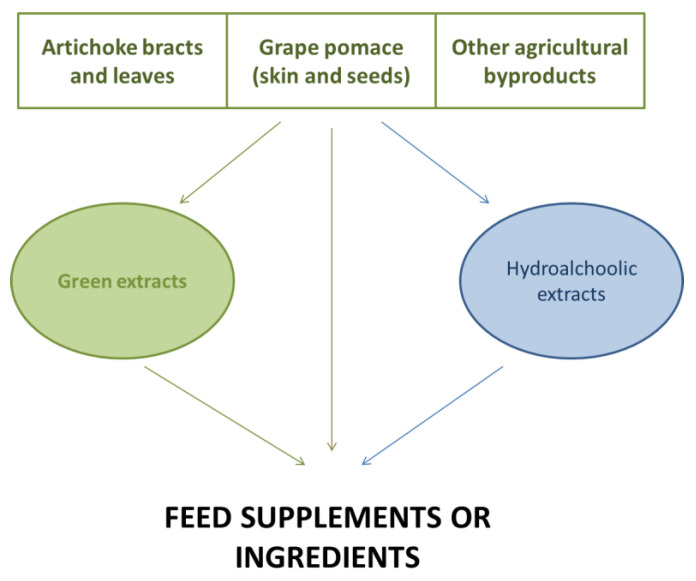
Different options for the production of animal feed supplements.

Recently, research on innovative and sustainable methods for the extraction of bioactive components from agro-food wastes has been carried out in order to produce animal feed supplements. In this paper, we aimed to review advances made in innovative extraction techniques which could have significant implications for the sustainability of future animal production (Figure 1).

## 2. Agricultural Waste as a Source of Antioxidants for Broiler Nutrition

### 2.1. Grape Waste Products

Grape (*Vitis* spp.) is one of the most widespread crops, with raw fruit and grape-based food products—mainly wine, but also juices, jams and vinegar—being incredibly important for human consumption. The winemaking process accounts for 75% of the total utilization of grapes, but it takes a heavy environmental toll, and it is estimated that about 0.3% of greenhouse gas emissions worldwide can be attributed to the wine industry. Moreover, vinification produces solid waste products, among which plant remains, grape pomace (GP) and wine lees are the most important [34]. Some of these byproducts, such as grape pomace, have garnered increasing interest as food and feed supplements over the last fifteen years [35,36,37,38,39]. Such waste could easily be harnessed for animal feed production, improving animal welfare and growth as well as meat quality, thanks to the presence of functional ingredients and bioactive constituents (namely polyphenols and dietary fiber) [40].

Grape pomace consists of grape seeds, skins and stems, and it represents about 20% of the total weight of grapes used for winemaking. In particular, GP is rich in flavonoids such as catechins and procyanidins, and its polyphenols are potent antioxidants capable of scavenging Reactive Oxygen Species (ROS). ROS activity was observed to improve oxidative stability in various raw meats, including beef, chicken, pork, lamb and fish [34,41]. Grape pomace extract has also shown promising activity as an anthelmintic agent, being capable of inhibiting the hatching, development and motility of sheep parasite *H. contortus* [42]. Moreover, the well-known antimicrobial properties of grape pomace extracts from several grape varieties and cultivars also make these vinification byproducts particularly attractive as feed supplements, capable of substituting antibiotics [43,44].

A recent study by Romero et al. [45] explored the effects of feeding broiler chickens with grape seed or grape skin meals, or with a combination of the two meant to simulate a reconstituted grape pomace. These three experimental diets were compared to a control (corn-soybean diet) and a control + vitamin E diet. While no effects of dietary treatments were observed on daily feed intake and on spleen weight, the dietary inclusion of grape skin meal (110 g/kg) increased feed conversion ratio by 15.6% compared to control and impaired chicken growth, inducing a reduction of daily weight gain by decreasing ileal protein digestibility. This effect, however, could not be attributed unequivocally to the presence of grape polyphenols, since the diet containing only grape seed meal did not provide a similar result, despite containing the same concentration of grape polyphenols (ca. 2.4 g/kg). Indeed, the results of this study would suggest that the different kinds of polyphenols contained in grape seeds and grape skin, and the different concentrations of nonextractable polyphenols from these two sources, are responsible for the different results obtained by the two diets. In particular, grape skins contain a lower concentration of proanthocyanidins, with a higher degree of polymerization compared to grape seeds, and they also feature a much higher concentration of non-extractable polyphenols. Finally, the combination of grape seeds and skin in the diet resulted in increased concentrations of antioxidants α- and γ-tocopherol in plasma and in 7-day stored raw meat, delaying lipid oxidation in thigh meat in a manner similar to the vitamin E-supplemented diet. These results suggested the need to assess the optimal proportions of grape seeds and skin in the diet of broiler chickens in order to maximize antioxidant activity without reducing growth rate [45]. Moreover, it was found that feeding of grape seed powder in broilers positively ameliorated the negative effects of *E. tenella* infection on growth performance, lesion score and oocysts shedding [46]. Additionally, grape seed improved growth performance, antioxidant status and immune response in broilers under heat stress conditions [47].

In another study, the effects of dietary supplementation with grape pomace (as raw ingredient) or grape seed extract were studied in chickens [40]. The concentrations of food-derived phenolic compounds were analyzed in the plasma and thigh meat of the broilers fed with the experimental diets. Thirty-two phenolic compounds were detected in plasma, and for 21 of them, significant differences were found between the two grape-based diets. Eleven of these metabolites were only detected in samples from chickens fed with grape seed extracts, while others were found at higher concentrations in one or both the grape-based groups, as compared to the control. Monomeric flavanols such as epicatechin and catechin were among these compounds; they were further metabolized into sulphate derivatives. Metabolites of polymeric flavanols, namely the sulphated form of dihydroxyphenyl-γ-valerolactone, were also detected in the experimental groups, meaning that at least a fraction of polymeric flavanols were transformed by the intestinal microbiota of chicken. The content of flavanol monomers was greater in chicken fed with grape seed extract supplementation as compared to the grape pomace group; the authors hypothesized that this difference may have been due to the fact that flavanols are free in grape seed extractm while a high proportion of them are called nonextractable proanthocyanidins [40]. Moreover, the high dietary fiber content of grape pomace may have affected the different fate of flavanol metabolism. This study showed the presence of circulating phenols in chickens fed with grape-based supplemented feeds, which could be interpreted as an improvement of the plasma antioxidant status of the birds. In thigh meat, no increase in phenolic metabolites was observed, although the possible antioxidant effects exerted by supplemented phenols during their long-term circulation through the organism remained unknown. Similarly, chicken fed with the experimental diets showed higher plasma concentrations of α- and γ-tocopherol, while, again no clear increase of these substances was detected in thigh meat [40].

In a recent work, dietary incorporation of grape pomace at the inclusion level of 2.5% as alternative to in-feed antibiotics was shown to exert beneficial effects on the growth performances of broiler chickens during the starter phase (days 1–14); from days 14 to 28, the diets showed slightly negative effects on growth performances. No differences in feed efficiency were found throughout the overall period [48]. GP inclusion improved the villus height and villus height crypt depth ratio and the modulation of gut microbiota, while the ceca short-chain fatty acid concentrations were not affected in birds fed grape pomace. Therefore, the inclusion of grape pomace at 2.5% in the diet of broiler chickens was favorable for the optimization of intestinal health without affecting their blood biochemical and immune profiles [48]. The impacts of GP, as reported in the literature, depended on its inclusion level in chickens’ diets; Goñi et al., and Sáyago-Ayerdi et al., reported that addition of dietary GP up to 6% did not impair growth performance. Moreover, supplementation of 5% or 10% GP did not improve growth performances of broiler chickens [49].

The dietary effects of vinification byproducts, such as grape pomace, grape stem extract and wine lees extract, have been studied with respect to their antioxidant and anti-inflammatory status in broilers [50]. No differences among the diets were observed with respect to growth performance, feed intake and feed conversion ratio. Moreover, while grape pomace had no effect on the chicken’s oxidative status, the other two polyphenol-rich extracts were found to significantly affect the expression of one of the NADPH oxidase enzymes (NOX3) in the liver, suggesting that the polyphenol composition profile of each extract was able to regulate in vivo antioxidant mechanisms. As for plasma, the catalase (CAT) and superoxide dismutase (SOD) activities were significantly increased in the grape lees extract-fed group in comparison with the grape stem extract one, thus reducing the level of ROS [50]. With regards to the broilers’ inflammatory state, no major differences were found between the control group and the three groups fed with vinification byproducts [50,51]. This study unveiled insights into a low-grade pro-inflammatory response when stem extracts were supplemented in the broiler diet under a transcriptional point of view. Thus, lees are considered to be a valuable winemaking byproduct, capable of improving broilers’ antioxidative mechanisms and muscle oxidative stability without adversely affecting birds’ performances and immune regulation [51].

The majority of catechins contained in GP are contained in polymeric structures, drastically lowering their bioavailability [52,53], and their absorption depends on the ability of the gut microbiota to hydrolyze polymers into monomers or oligomers [54,55,56,57]. This is a significant obstacle to the use of GP as a food supplement, which was tackled in a recent study [58]. In particular, this study tested the effects of two levels of GP (5 and 10%) with or without tannase (T) or an enzymatic complex (EC) or both (T + EC) on the intestinal utilization of the polyphenolic compounds present in GP and on the structural changes obtained with the inclusion of enzymes. The chicken antioxidant status was also investigated. The ileal protein digestibility was significantly higher when GP was included at 5% in comparison with 10%, and this parameter was negatively affected by the addition of tannase, alone and in combination with the enzymatic complex. No effect was observed when the EC was added alone. Similarly, birds fed with 5% GP showed a higher total polyphenol digestibility than those fed 10% GP, with or without T and EC addition, alone or in combination. This approach, however, resulted in a reduction of intestinal catechin absorption, suggesting that the presence of polymeric species might contribute to the absorption of oligomers and monomers [58]. At any rate, GP supplementation was responsible for increased plasma α-tocopherol concentrations and reduced plasma iron content, further confirming this supplement’s ability to improve the antioxidant status of broilers [58].

### 2.2. Artichoke Waste and Bracts

Artichoke (*Cynara scolymus* L.) has been used as food and as a traditional remedy since ancient times due to its high quantities of bioactive compounds [59,60,61]. Much like the winemaking industry, the industrial processing of artichokes also generates large amounts of waste biomass [60,61,62,63,64]. These discarded materials still contain a large variety of bioactive compounds endowed with therapeutic properties [61]. The heart and inner leaves of this vegetable are considered edible, whereas the external bracts, leaves and stems are non-food industrial byproducts. Importantly, these byproducts represent about 60% of the artichoke mass [65,66]. Indeed, leaves, stems and most especially the external bracts are rich in phenolic compounds, flavonoids (apigenin and luteolin) [67], sesquiterpene, fiber, bioactive carbohydrates (inositols and inulin) and minerals necessary for human nutrition, with antioxidant, hepatoprotective, diuretic, antifungal, antibacterial and anticarcinogenic effects [65,67,68]. Inositols are mainly found in vegetables, thus their presence in animals and meat is limited [65]. Inulin is a reserve carbohydrate with recognized prebiotic properties, and it is used as a technological ingredient [65]. Artichokes are also considered a rich source of vitamins C and K, as well as of microelements such as calcium, iron and zinc [67]. The byproducts extracted from artichokes can be used as fiber supplements in the functionalization of products [64] or they can be used as functional food or feed additives for animals [69,70]. Artichoke bracts are a good source of bioactive molecules. Therefore, it is necessary to identify sustainable extraction methods able to valorize these wastes and to exploit their interesting compounds [60,71].

Artichoke leaves are another important source of bioactive molecules that could be useful for supplementation in poultry feed. Several papers reported that the use of *C. scolymus* increased the body weight and feed intake of broilers in most cases without significant effects on the feed conversion ratio. Moreover, these artichoke-based supplements did not affect broiler carcass characteristics. The main effect of such supplementation seemed to be a reduction in circulating levels of cholesterol and triglycerides, contributing to lower blood pressure. *C. scolymus* can be used to improve the health status, since it ameliorates the immune system response of broilers; as a matter of fact, an increase in Newcastle antibody titers in vaccinated chickens was also found [72].

## 3. Extraction Methods of Antioxidants from Food Waste: Towards a Greener Approach

As seen in the previous paragraph, the large amount of waste generated by the food industry represents a precious resource for society; these wastes can be harnessed for the functionalization of animal feed and human food supplements [18]. Moreover, many of the previously discussed studies showed that the extraction of the bioactive components from these sources was often preferable to their use as-is. In this section, the main extraction techniques from agricultural waste that could help harness it for animal nutrition were discussed, as were the most recent innovations in this area.

### 3.1. Green Extraction Methods of Antioxidants from Grape Pomace

The extraction of antioxidants such as polyphenols from GP has been a topic of interest for the past two decades [73,74,75,76,77,78,79,80,81,82]. Although simple hydroalcoholic extraction methods (which themselves can be categorized as green) are the most commonly used for the extraction of polyphenols and other bioactive compounds [73,74,75,76,77,78,79,80,81,82], the past decade has seen remarkable developments in the optimization of these methods (e.g., through the use of microwaves, pressurized solvents or ultrasounds) [83,84,85,86,87,88,89,90,91,92,93]. Moreover, completely new solvents, such as Deep Eutectic Solvents (DESs), have been introduced into this field, providing several promising advantages over classical green extraction procedures [94,95,96,97,98,99,100].

Among hydroalcoholic extraction methods, a mechanical orbital agitation was recently optimized in a methanol-water 80:20 (*v*/*v*) solution [77]. In particular, plate agitation, orbital agitation and ultrasound were compared, with the first two methods being more efficient than the latter for the extraction of total polyphenols. Plate and orbital agitation showed significant differences regarding the extraction of most phenolic compounds, except for gallic acid, epicatechin, myricetin and quercetin [77]. As expected, extraction efficiency was directly related to total extraction time and agitation speed; however, long extraction times can result in the degradation of phenolic compounds, as well as generating higher energy costs [77]. For this reason, orbital agitation (5 min) was preferable to plate agitation (120 min). Furthermore, the authors evaluated the effects of different solvents in the hydroalcoholic mixture, thus comparing 80% solutions of methanol, ethanol and acetone. Among these, methanol was shown to be the best solvent for the extraction of polyphenols, especially for substances such as trans-resveratrol, although the levels of syringic acid, ferulic acid and peonidin-3-*O*-glucoside were significantly lower compared to the other two extracts [77].

A similar study was carried out on grape pomace from the Cabernet Sauvignon, Merlot and Italian Riesling varieties, comparing the extraction efficiency of several hydroalcoholic solutions with that of other organic solvents such as acetone and ethyl acetate [78]. The quantity of selected polyphenols and triterpenoid compounds in each extract was evaluated via liquid chromatography (LC/MS-MS), and while the ethyl acetate extract was shown to be the most enriched with the desired polyphenols (especially flavanols, coumarins and stilbenes), methanol and acetone-based solvents were more appropriate for the extraction of anthocyanins [78].

The optimal solvent for polyphenol extraction seems to vary according to the grape variety. Indeed, a study carried out on grape pomace from the Nebbiolo variety showed that an aqueous solution of acetone was the most efficient extraction solvent [79]. This method was later applied by the same authors to vinification byproducts from four different cultivars typically grown in the Italian Piemonte region (namely Nebbiolo, Uvalino, Barbera and Albarossa), taking into account the separate components of GP (namely grape seeds and skins) [80]. In a recent study, the characterization of ethanolic extracts from GP samples obtained from *Vitis vinifera* (specifically the Cabernet Sauvignon and Merlot varieties) and *Vitis labrusca* (Terci variety and a mix containing Bordeaux 65%, Isabel 25% and BRS Violet 10%) was carried out via HPLC-DAD and HPLC-MS/MS [81]. In particular, the Terci variety showed the highest content of anthocyanins, followed by the mixed sample, the Cabernet Sauvignon and finally, Merlot. Hydroxybenzoic acids were most concentrated in the mixed sample and in the Terci sample; in detail, gallic and syringic acid were more highly represented in the mixed sample, while the Terci sample showed the highest concentration of vanillic acid. Among hydroxycinnamic acids, caffeic acid was the only compound detected in the Cabernet Sauvignon sample, while *p*-coumaric, chlorogenic and caffeic acids were more abundant in the mixed sample; at the same time, the Terci variety showed the highest concentrations of *trans*-cinnamic acid. Flavanols such as catechins were also widely represented among these samples, with the mixed sample possessing the highest concentrations of catechins, while flavonols such as quercetin and kaempferol were most concentrated in the Merlot sample. As expected, *trans*-resveratrol was present in all samples, with the highest concentration in the Merlot one [81]. An extract obtained with the same extraction method from Merlot grape pomace was reported to ameliorate the oxidative stress conditions in a rat model of arthritis via the involvement of anthocyanins, as well as alleviating inflammation through the activity of catechin and epicatechin derivatives, which are known for their anti-inflammatory activity [82].

A different method, involving exhaustive maceration for 7 days of 10 g of powdered Máximo IAC 138–22 grape pomace in 100 mL of ethanol 70%, was applied for the extraction of polyphenols. The gross extract showed anthelminthic activity in sheep—and its serial partitioning in volatile organic solvents of increasing polarity, such as hexane, dichloromethane and ethyl acetate, followed by a final extraction in water, resulted in a significant decrease in its bioactivity [42].

However, organic solvents are not strictly necessary for these procedures. Indeed, a procedure involving only water was recently optimized for the extraction of grape pomace from the Montepulciano cultivar [101]. Characterization of this extract was carried out via HPLC and highlighted the presence of gallic acid, caftaric acid, catechin, chlorogenic acid, epicatechin, caffeic and syringic acid. This extract also proved to be nontoxic at a concentration of 10 mg/mL in the *Daphnia magna* toxicity model and showed significant antioxidant and protective properties in HypoE22 cells. The same extract inhibited the H_2_O_2_-induced expression of COX2 in the same cell line, resulting in a lower production of prostaglandin E2 [101].

Although hydroalcoholic solutions have proven to be excellent for the extraction of polyphenols, a significant improvement in the extraction efficiency was achieved with novel methods involving pressurized liquid extraction (PLE). The effects of ultrasounds and microwaves have also attracted significant attention for the same reasons. These methods are highly advantageous when compared to conventional liquid extraction, since they provide higher extraction yields in a lower time along with a lower amount of solvents. Indeed, the past ten years have seen a continuously rising interest in these methods [83,84,85,86,87,88]. 

In a study, the results of classic solid-liquid extraction (in a 50% *v*/*v* ethanol–acidic water solution) of grape pomace from the Tempranillo variety were compared to those obtained by the same method after a microwave-pressure pretreatment. Total anthocyanins were measured via the pH-differential method, while phenolic metabolites (namely gallic acid, catechin, procyanidin B2, epicatechin, myricetin, quercetin and malvidin) were detected by HPLC-DAD. Microwave pretreatment of the sample gave a higher polyphenol yield in a shorter time, also improving the concentration of anthocyanins in the sample up to 85% [89]. 

Likewise, ultrasounds have allowed a better extraction of polyphenols from grape pomace. In fact, in a recent work, grape pomace from an unspecified cultivar of *Vitis vinifera* was extracted in a hydroalcoholic medium (50% ethanol) with an ultrasound-assisted procedure [90]. Several extracts were obtained at different extraction temperatures (25, 45 and 65 °C) and were sampled at intervals of 5 min to 30 min. The authors then compared the concentration of total anthocyanins (spectrophotometrically determined) in this extract to the one obtained by conventional solvent extraction. Notably, the benefits of ultrasounds were higher in the beginning of the extraction and quickly decreased over time. Moreover, the authors of this study developed two mathematical models for the extraction of anthocyanins from grape pomace, one for conventional solvent extraction and another for UAE [90].

Similarly, another work was conducted on UAE from fresh and oven-dried Tempranillo grape pomace, seeds and stems [91]. In this case, response surface methodology (RSM) showed that the optimal extraction conditions for total polyphenols (measured by Folin–Ciocalteu assay) could be achieved with 44% ethanol solvent, at 25 °C for 3 min, in two extraction cycles. The presence of polyphenols (in particular flavan-3-ols, stilbenes, flavonols, hydroxycinnamic acids and anthocyanins) were determined via HPLC-DAD-MSn. Fresh grape pomace was found to be the richest sample of all the studied polyphenols; however, oven-drying was responsible for a significant decrease in total anthocyanins [91].

The previously reported method was compared to a PLE (aqueous solution, 120 °C, 1500 psi, in two extraction cycles of 10 min each) in a subsequent study [92]. Grape pomace samples (seeds, stems and whole pomace) from Tempranillo and Cabernet Sauvignon grape seeds were extracted and their phenolic profile was spectrophotometrically evaluated. UAE resulted in a significantly higher phenolic index compared to PLE; in detail, hydroxycinnamic acid derivatives and flavonols were less concentrated in the UAE, while tannins, catechins and anthocyanins were more concentrated. The concentration of these species was directly correlated with the antioxidant and antimicrobial activity of these extracts [92].

Further optimizations of the UAE methodology have been achieved by combining it with conventional solvent extraction, carrying out the extraction of freeze-dried Cabernet Sauvignon grape pomace in water or in 50% EtOH at 25, 35 or 45 °C. Total phenolic content (TPC) increased with higher temperatures and with application of conventional shaking between intervals of ultrasound treatment, and the hydroalcoholic solvent displayed a higher extraction capacity than the aqueous solution [93].

It must be highlighted that while alcohols, such as ethanol and methanol, can be considered green solvents due to the low environmental impact of their disposal [102], their toxicity to animals means that their use in feed and food supplement requires their complete removal, which can be an energy-intensive procedure. Thus, research has also focused on developing new, non-thermal extraction methods allowing the use of water as only solvent or of non-toxic solvents such as DESs [94,95,96,97,98,99,100,103,104].

Pulsed electric discharge (PED) is another innovative, non-thermal extraction technique based on the formation of pores in the plant cell caused by forceful electric discharges. A recent paper described a continuous extraction method using a needle-ring design for the extraction chamber, with comparable extraction efficiency to previously known PED methods, but with the significant advantages of shorter extraction times and lower energy consumption [103]. 

Alternative solvents have also been explored. For example, a study carried out a few years ago reported the optimization of an extraction method using aqueous glycerol-tartaric acid solutions at different concentrations by RSM [104]. Both substances were natural compounds, with low costs and no toxicity. In particular, this study proved that 20% glycerol (*v*/*v*) in water could achieve optimal levels for polyphenol extraction, while adding tartaric acids had overall negative effects [104]. 

Glycerol and tartaric acid are also very common components of DESs, in which they act as hydrogen bond donors. DESs are an innovative solution to the energetic shortcomings of classical solvents and several studies have proven their excellent capacity for extracting a variety of bioactive substances from grape pomace [94,95,96,97,98,99,100]. When using these novel solvents, the first step is to determine the optimal combination of DESs components and the ratio between them. A recent work featured a comparison of the extraction efficiency of flavan-3-ols, anthocyanins and flavonols from red grape skin of several DESs (namely ChCl:Gly 1:2, ChCl:Oa 1:1, ChCl:Ma 1.5:1, ChCl:Sor 1:1 and ChCl:Pro:Ma 1:1:1) [94]. Each DES was tested at 0, 10, 25 and 50% water in order to evaluate the effects of their viscosity on extraction efficiency, and the extracts were obtained in a shaker, with an extraction time of 12 h at room temperature. The extraction efficiency of these DESs was also compared to that of two different methods with water or hydroalcoholic solutions, namely water:MeOH 70:30 and water:MeOH:12 M HCl 70:29:1. HPLC-DAD analysis was used to identify the extracted polyphenols. While overall extraction efficiency was highly dependent on water content, each DES showed a different capability to extract polyphenol species; for instance, the ChCl:Oa extract demonstrated the highest concentration of total anthocyanins, followed (in order) by ChCl:Ma, ChCl:Ma:Pro, ChCl:Gly and, finally, ChCl:Sor. This result was not entirely unexpected, due to the fact that anthocyanins are extracted more easily the more a solvent is polar and acidic [94]. Moreover, ChCl:Oa with 25% water proved to be the best overall solvent in regards to anthocyanins, showing a 5-fold increase in the content of total anthocyanins compared to water and a 2-fold increase compared to methanol:water 70:30 (*v*/*v*). Moreover, this extract also showed a higher concentration of each individual anthocyanin, and the highest concentrations of (+)-catechin in regards to which all DESs were superior to conventional solvents. This was also true of quercetin-3-*O*-glucoside, similarly to traditional solvents. Furthermore, this promising DES was used in UAE and MAE procedures in order to optimize extraction conditions by reducing energy consumption and extraction time. UAE proved to be a better extraction method than MAE, with a temperature of 65 °C and an extraction time of 50 min as optimal conditions [94].

The properties of components of DESs, such as HBD or HBA, must be taken into consideration when studying their interaction with an extraction matrix. As an example, two glycerol-based DESs were formulated with different HBAs (sodium acetate and sodium benzoate, SAc and SBz, respectively) in order to explore the effect of HBA polarity on extraction efficiency [95]. Thus, the two DESs (Gly:SAc 9:1 and Gly:SBz 9:1) were used for extraction after being diluted with water at a concentration ranging from 10% to 40% (*w*/*w*). While the optimal water percentage was 30%, the Gly:SBz DES consistently outperformed the Gly:SAc one in terms of total extracted polyphenols. The potential of this extraction method was further explored by coupling it with ultrasonication, achieving higher levels of total polyphenols in both DESs [95]. However, polyphenols were found to be more soluble and stable in Gly:SBz. Compared to aqueous and hydroalcoholic extracts, the Gly:SBz extract showed significantly higher concentrations of several polyphenolic metabolites, namely gallic acid, caftaric acid, catechins, rutin and quercetin; anthocyanins, especially malvidin-3-*O*-glucoside and its *p*-coumarate ester were also present at higher concentrations in the DES [95].

In another recent study, DESs based on betaine (Bet) and ChCl combined with ethylene glycol (EG), citric acid (Cit) and urea were used to obtain grape pomace extracts that were subsequently tested for their antioxidant activity and inhibition of urease. In particular, ChCl:EG showed the best antioxidant potential and the highest effect in urease inhibition [96]. The DES extracts (mainly EG-based DES) and their polyphenol formulations, tested at a concentration of 34 mM of CHO or BET, showed very low phytotoxicity on cress seedlings and on the early growth of oat. On the other hand, tests performed on earthworms showed that CHO-based DES could impair their reproduction, and U-based DES caused severe mortality [96].

NADESs have also been employed in the extraction of polyphenols from grape pomace. For instance, a procedure involving a ChCl:Cit (2:1, 30% water) NADES in an ultrasonic-microwave cooperative reactor proved able to determine higher levels of anthocyanins, gallic acid, (+)-catechin and quercetin-3-*O*-glucoside compared to those obtained with an aqueous solution of ethanol (70%) [97]. The biological activity of the NADES extract (tested as-is, without solvent removal) was also evaluated, showing antioxidant properties and antiproliferative effects on cancer cell lines [105]. The same authors further optimized this procedure up to 0.5 L batches; other NADESs were also evaluated with ChCl:Cit 2:1 still showing the best results [97].

Several other NADESs, based on Bet as HBA and with urea, Cit and EG as HBDs, were also used to extract polyphenols from grape pomace [98]. The contents of each extract were analyzed for the presence of anthocyanins by HPLC-MS/MS. In detail, all the solvents showed a particular selectivity to malvidin, and the Bet:EG extract showed comparable results to hydroalcoholic mixtures, while the other two NADESs possessed higher extraction abilities compared to hydroalcoholic solvents [98]. More NADESs were tested and used in combination with pressurized hot water extraction. After such a screening, a ChCl:Oa DES, with 30% water and an extraction temperature of 60 °C, was selected as the optimal solvent for anthocyanin extraction from grape pomace [99].

From all these studies, it is evident that the extraction efficiency (and thus the advantageous profile) of DESs and NADESs is strongly linked to their composition. For this reason, a computational method (COSMOtherm) for their selection was recently applied [100]. Although COSMOtherm was not originally developed for DES optimization, its predictions regarding the concentration of polyphenols in several DESs that were screened as part of this work were accurate and allowed researchers to identify combinations, such as Betaine:Glucose 1:1 or Betaine:Sucrose 1:1, that were optimal for polyphenol extraction [100].

### 3.2. Green Extraction Methods of Antioxidants from Artichoke Wastes

Over the years, several techniques have been developed to extract bioactive compounds from artichoke waste products. For example, D’Antuono et al. [67] used parts of artichokes that were not suitable for the market, like undersized artichoke heads. Artichoke heads were homogenized and a mixture of food grade solvents was used for the extraction of bioactive compounds by maceration. Of these, the mixture ethanol:water 80:20 (*v*/*v*) turned out to be the best extracting solvent. Finally, HPLC-DAD analysis was used to identify the main polyphenols present in the hydroalcoholic extract. Mono- and di-caffeoylquinic acids were identified in the extract, but the results highlighted the presence of chlorogenic, 3,5-*O*-dicaffeoylquinic and 1,5-*O*-dicaffeoylquinic acids as the most concentrated compounds in artichoke heads [67]. 

In the last years, non-conventional extraction techniques, such as PLE, MAE and supercritical fluid extraction (SFE), have also been applied for the extraction from artichoke wastes [61,63,71]. In particular, PLE and MAE have been applied to the extraction of inositols and inulin from artichoke bracts. MAE was found to be more effective for the extraction of bioactive cyclitols, whereas PLE provided higher yields of inulin. In a recent work, Mena-Garcìa et al. [71] developed a new methodology based on MAE for the simultaneous extraction and analysis of low molecular weight carbohydrates and caffeoylquinic acids from artichoke byproducts. MAE parameters were chosen in order to maximize the antioxidant activity and the concentration of caffeoylquinic acids, inositols and total phenolic compounds [71]. The effect of different solvents, extraction times and temperatures were studied; the most suitable solvent for the extraction of inositols and phenolic compounds was MeOH:H_2_O (50:50, *v*/*v*) and the optimal extraction temperature and time were 60 °C and 3 min, respectively. However, EtOH:H_2_O (50:50, *v*/*v*) was investigated further, considering that this solvent is less toxic and more environmentally friendly than the MeOH:H_2_O mixture. The optimal MAE conditions for the extraction in ethanolic solution of bioactive compounds were 98 °C and 3 min. GC-MS analysis showed the presence of caffeoylquinic acids in bracts and receptacles. In particular, the highest concentration of chlorogenic acid was found in the receptacles sample extract, while caffeic acid was only detected in bracts and receptacles extracts [71]. 

In a further application of hydroalcoholic solvents, the extraction of phenolic compounds from artichoke wastes in water was compared to the same procedure carried out in a MeOH:H_2_O (60:40, *v*/*v*) mixture, with and without ultrasound application [61]. Analyses of each fraction were performed via HPLC-DAD, showing significant differences (both qualitative and quantitative) in the phenolic profile of each extract, depending on the extraction method used. The richest extract in phenolic compounds was obtained using MeOH:H_2_O (60:40, *v*/*v*) with ultrasound application. Methanol was much more efficient than water for extracting both hydroxycinnamic acids and flavonoids, but caffeic acid was not detected in any sample. The most abundant flavonoids in the extract obtained from artichoke waste using 60% MeOH were luteolin-7-*O*-rutinoside and luteolin-7-*O*-glucoside. In the extract using water as a solvent, the most extracted flavonoid was luteolin-7-*O*-rutinoside, although the extraction efficiency was much lower than when methanol was used [61]. The ultrasound application increased the content of phenolic compounds when 60% methanol was used as the solvent. Ultrasound normally improved the extraction of bioactive compounds and did not produce any significant changes in the properties and functionality of most of the bioactive compounds—making it ideal for the extraction of antioxidants. The antioxidant capacity of the extracts obtained using 60% MeOH as the extraction solvent was much higher than that observed in the extracts obtained with water. On the other hand, the antioxidant capacity of the extracts obtained from UAE was very similar to that obtained by single solvent extraction [61]. 

In a recent study, Francavilla et al. [63] investigated two cascading extraction methods based on MAE in hydroethanolic mixtures, aiming to improve temperature, solvent and extraction time conditions. An aqueous solution, a 100% EtOH solution and three different EtOH/H_2_O mixtures (25, 59 and 75%, *v*/*v*) were used at three different temperatures (50, 75 and 100 °C) with three different extraction times (5, 10 and 20 min) [63]. The process, designed for valorizing different globe artichoke plant residues, was conducted in two steps. In the first step, phenols were extracted from raw biomass generating a marketable product (phenolic extract) and a byproduct called phenolic-extracted residue (PER). In the second extraction process, PERs were themselves extracted, producing a further marketable product (inulin) and a byproduct named inulin-extracted residue (IER), which has been characterized for its possible valorization as bioenergy feedstock and for agricultural application (green manure). Pure water and pure ethanol were the worst solvents, while ethanol 25% was the best [63]. However, EtOH concentrations higher than 60% generated a decrease in the extraction yield and polyphenol recovery. In conventional extraction, without microwave, the highest yields were found using EtOH/H_2_O (50:50, *v*/*v*) and EtOH/H_2_O (75:25, *v*/*v*). Moreover, the effects of extraction time and temperatures were studied as well. As such, the best extraction conditions for MAE polyphenols extraction were: solvent EtOH 25%, time 5 min, temperature 50 °C. Chlorogenic acid (5-*O*-caffeoylquinic acid) was the most abundant component, followed by 1,5-*O*-dicaffeoylquinic acid [63]. These results were comparable with those reported in another paper [71]. For inulin extraction, the best reported conditions were MAE, EtOH 25%, 5 min and 80 °C. Inulin content was impressively higher than that reported by other authors [106] for the same artichoke cultivar (12% dw) extracted by ultrasound-assisted extraction (UAE) in water at the same temperature [63]. These results were also better than those obtained in a previous study that investigated inulin extraction from artichoke’s external bracts using MAE. In fact, under the best MAE conditions (120 °C, 3 min), a lower inulin yield was achieved [65], compared to the results obtained by Francavilla and colleagues [63].

While the extraction of bioactive compounds from artichoke waste with hydroalcoholic solutions has been thoroughly investigated, no research has been carried out so far on ways to obtain similar extracts with DESs, which would be more advantageous for animal feed and the formulation of food supplements. 

### 3.3. Green Extraction Methods of Antioxidants from Other Vegetable Sources

Agricultural waste goes beyond just vinification and artichoke byproducts. Indeed, modern agriculture produces large amounts of organic waste which, if not properly utilized, accumulate in landfills [107]. Thus, their use as sources of feed and food supplements would turn these accumulating wastes into a precious resource. This has captured the attention of researchers—indeed, in the last few years, a remarkable amount of research has been carried out on the use of novel methods and DESs for green extraction procedures from all sorts of agricultural waste. 

Olive tree (*Olea europaea*) leaves contain antioxidant compounds. For this reason, Kırbaşlar et al. [108] investigated MAE with different conventional solvents (water, acetone, acetonitrile, methanol and ethanol) at different concentrations, and evaluated total phenolics (TPI), flavonoids (TFI) and antioxidant activity (AA) of extracts [108]. Once it was determined that the best concentration was 30% by volume, other parameters, namely microwave power, extraction time and total solvent volume, were investigated. In general, the AA increased with the increase of TFI and TPI [108]. On the other hand, Yücel et al. [109] developed an eco-friendly method to produce an oleuropein-rich extract from Olive tree (*Olea europaea*) leaves. Oleuropein is a polyphenol known for its beneficial antioxidant and antibacterial effects. Many DES mixtures were tested combining a carboxylic acid as HBA and a polyol or a salt as HBD at different ratios using homogenizer-assisted extraction (HAE). TPM and oleuropein content in the extracts measured by UV-visible spectrometry and HPLC revealed that glycerol/lactic acid (Gly/La) 1:1 was the best DES [109]. This mixture was used for extraction optimization. In particular, the influence of parameters such as water content, speed and time on antioxidant activity were studied, through DPPH, ABTS and CUPRAC assays. The best results were obtained by 50% aqueous DES Gly/La 1:1 (mol/mol), as confirmed by RSM. Finally, this extract was stored at different temperature conditions, and it was found to be stable for two months at room temperature and up to six months in colder environments [109].

Green tea (*Camellia sinensis*) is rich in antioxidant polyphenols, in particular catechins. Luo et al., studied a green UAE-DES extraction method. Among twelve ChCl-based DESs, a ChCl-glycerol mixture was selected for optimization [110]. Response surface methodology was used for investigating the influence of various parameters, such as ultrasonic power and time, on the TPC. Moreover, the obtained extract showed higher antioxidant properties than conventional methods UAE-ethanol, ethanol and hot water extraction, evaluated though ferric-reducing antioxidant power (FRAP), 1,1-diphenyl-2-picrylhydrazyl (DPPH) and diammonium salt (ABTS) assays. In addition, the quantification by HPLC revealed that major catechins in green tea extract were (−)-epicatechin (EC), (−)-epigallocatechin (EGC), (−)-epicatechin gallate (ECG) and (−)-epigallocatechin gallate (EGCG). Finally, scanning electron microscopy (SEM) analysis demonstrated that UAE-DES efficiency in polyphenol extraction was probably due to the surface erosion of green tea leaves, which allowed a better penetration of the solvent [110].

Kiwi (*Actinidia deliciosa*) is rich in antioxidant nutrients, such as polyphenols, carotenoids and vitamin C. Giordano et al., described an ultrasound-assisted extraction method of flavonoids from kiwi peel. HPLC-DAD-ESI/MSn analysis revealed that principal flavonoids in the extract were quercetin-3-*O*-glucoside and quercetin-3-*O*-ramnoside as quercetin glycosides, with epicatechin and a B-type (epi)catechin dimer as flavan-3-ols. RSM was performed to study the effects of three individual variables (extraction time, ultrasonic power and ethanol concentration) on flavonoid content and extraction yield [111]. 

Under the optimized overall UAE condition, kiwi peel extract was found to have antioxidant, anti-inflammatory and antimicrobic activities, with no cytotoxic effects, as evaluated in vitro [111].

A ChCl-based DES was studied for the extraction of phenolics from rosemary leaves (*Rosmarinus officinalis* L.) as well [29]. Rosemary is a food additive with antioxidant and antimicrobial properties. Ten ChCl-based DESs (neat and with the addition of water) were screened for the extraction of phenolic compounds from rosemary leaves. Conductor-like screening model for realistic solvatation (COSMO-RS) method, used for the prediction of the solubility of molecules in different solvents, confirmed experimental results: ChCl:1,2-propanediol (CPH) mixture was selected for optimization in solid-liquid extraction. This extract exhibited good antioxidant and antimicrobial properties [29].

Interestingly, bioactive compounds from rosemary leaves were also extracted using edible oils. These solvents have the advantage of being nontoxic. They also contain micronutrients, such as phospholipids and sterols [112]. TPC quantified by Folin–Ciocalteu test revealed that, among 12 tested oils, soybean oil was the most efficient. Regarding nonvolatile compounds, all 12 tested oils were capable of extracting apolar carnosol and carnosic acid but not polar rosmarinic acid. Moreover, the addition of oil derivatives, soy lecithin in particular, surprisingly improved the extraction efficiency of more polar compounds and volatile aroma compounds (VACs) on the basis of the original oleo-extraction. On the other hand, VACs were identified in all oily extracts, with different compositions. In conclusion, refined soybean oil with 1% (*w*/*w*) of soy lecithin was the best extraction solvent, as predicted by the above-cited COSMO-RS simulation [112].

Orange (*Citrus sinensis* L.) peel is rich in polyphenolic bioactive compounds, and a recent study focused on solid-liquid extraction (SLE) using DESs instead of volatile organic solvents [113]. ChCl was mixed with glycerol or ethylene glycol (EG,) in different ratios, and at different temperatures and extraction times. This optimization was monitored with the Folin–Ciocalteu method for the quantification of TPC and DPPH method for the determination of antioxidant ability of the obtained extracts. Results suggested that the best DES mixture was ChCl:EG 1:4 [113]. Moreover, a quali-quantitative determination of phenols contained in extracts was performed by RP-HPLC, confirming that ferulic acid was the main antioxidant, followed by *p*-coumaric acid and gallic acid [113].

Onion (*Allium cepa* L.) solid wastes (OSWs) are produced in very large quantities by European food industries; however, they contain a rich amount of antioxidant derivatives of quercetin [114]. For this reason, a recent work presented the optimization of a green extraction method based on DESs [114]. A mixture of sodium propionate as HBA and (L)-lactic acid or glycerol as HBD was used; results showed that the best DES was glycerol/sodium propionate with the ratio of 8:1. Moreover, the extraction could be performed at 80 °C without affecting the polyphenol yield or antioxidant properties of the extract. The extract obtained under these conditions could be also stored for 30 days at room temperature [114].

*Osmanthus fragrans* flowers exhibit various biological properties; in particular, they are used as antioxidant food ingredients [115]. While volatile components of *O. fragrans* flower oils are used to produce perfumes, non-volatile wastes, still rich in antioxidants (mainly flavonoids and phenolic acid), can be extracted and used as additive in food and cosmetics [115]. Pan et al., analyzed a microwave-assisted deep eutectic solvent extraction (MA-DESE) to obtain these extracts. Different DESs were prepared and used for the extraction, and radical scavenger assays (DPPH, ABTS and FRAP) were performed to determine the antioxidant activity of the so-obtained extracts. Results suggested that the antioxidant activity of DES-derived extracts was higher than that obtained by using the conventional solvent ethanol [115]. The optimization of the method was carried out on the best DES of the series, Glucose:La 5:1. Finally, the identification of the different components contained in DES Glucose:La extract, combined with microwave and 50% ethanol extract was performed by UPLC-MS. Interestingly, the content of flavonoids and phenolic acids in the DES extract was greater in comparison with the one of the ethanol extract, confirming results of the antioxidant activity assays [115].

The plant *Crocus sativus* is mainly used to produce the expensive spice saffron from the stigmas; the rest of the flower is usually wasted [116]. However, petals of the plant contain several polyphenols, such as anthocyanins and flavonol glycosides. Lakka et al., tried to extract these compounds by DESs. (L)-lactic acid and glycine were chosen as HBD and HBA, respectively; the optimal molar ratio was determined in 5:1. Subsequently, the extraction was optimized with RSM, while temperatures higher than 50–60 °C led to lower extraction yields [116]. At the end, the analysis of the polyphenols composition in the extract performed by liquid chromatography diode array mass spectrometry (LC-DAD-MS) revealed that the predominant flavonol was kaempferol 3-*O*-sophoroside, while delphinidin 3,5-di-*O*-glucoside was the main anthocyanin [116].

## 4. Conclusions

The present review focused on the enormous potential of agricultural wastes, including vinification byproducts and artichoke wastes, as a source of bioactive compounds obtained through different extraction methods. The best extraction conditions of the relevant studies reviewed in the text are summarized in Table 1.

In particular, bioactive molecules extracted from agricultural wastes may be interesting as supplements for animal feed. However, most polyphenol extraction is carried out by means of alcohol-based solvents in combination with techniques using ultrasound or microwaves to improve the bioactive compound extraction yields. Moreover, many of the studies reviewed herein presented notable differences in the characterization of extracts, in terms of their biological properties, when assessed. It is also quite difficult to compare different extraction methods due to the lack of standard characterization procedures.

Among the main critical points which limits the formulation of feed using such extracts is the wide use of alcohol-based solvents, which are toxic if introduced in supplements.

The identification of green solvents, such as DESs, may be useful, as they are nontoxic, edible and economically feasible. Furthermore, some components of these green solvents are already employed in animal feed as ingredients. The use of extracts as-is could be an intriguing goal for researchers, as well as an important breakthrough for industries.

## Figures and Tables

**Table 1:** List of the extraction conditions.

Starting Material	Solvent/Method	T (°C)	Extraction Time	Ref.
Non-pomace residue (*Vitis labrusca* L.)	MeOH:H_2_O 80:20, orbital agitation	25	5 min	[77]
Grape pomace (*Vitis labrusca* L.) (*Vitis vinifera* L.)	40% EtOH, shaking	25	24 h	[81,82]
Grape pomace (*V. vinifera* L.)	80% MeOH + acid	RT	6 h	[78]
Fermented grape pomace (*Vitis vinifera* L.)	Acetone:H_2_O 1:1 sonication	RT	20 min	[79,80]
Grape pomace (*V. vinifera* L.)	H_2_O	50	30 min	[101]
Fresh and oven-dried grape pomace Fresh and oven-dried stems Fresh and oven-dried seeds (*V. vinifera* L.)	44% EtOH, UAE	20	4 min	[91]
Grape pomace Grape stems Grape seeds (*V. vinifera* L.)	44% EtOH, UAE	20	4 min	[92]
	H_2_O, ASE (1200 psi)	120	2 × 10 min	
Grape pomace (*V. vinifera* L.)	70% EtOH Ultrasounds	RT	20 min	[42]
Grape pomace (*V. vinifera* L.)	50% EtOH/Acidic water Microwave-pressure pretreatment	100–60	120 s pretreatment, 3 h extraction	[89]
Grape pomace (*V. vinifera* L.)	50% EtOH Ultrasounds	45	400 s	[90]
Freeze-dried grape pomace (*V. vinifera* L.)	50% EtOH Ultrasounds	55	20 min	[93]
Oven-dried grape pomace (*V. vinifera* L.)	30% EtOH PED	RT	6 min	[103]
Grape pomace (*V. vinifera* L.)	20% glycerol/water (*v*/*v*)	RT	3 h	[104]
Grape skin (*V. vinifera* L.)	ChCl:Oa DES, 25% water, UAE	65	50 min	[94]
Grape pomace (*V. vinifera* L.)	Gly:SBz DES, 30% water, UAE	80	240 min	[95]
Grape pomace (*V. vinifera* L.)	Bet:U DES	RT	24 h	[96]
Grape pomace (*V. vinifera* L.)	ChCl:Cit DES, 30% water, UAE	65	50 min	[97]
Grape pomace (*V. vinifera* L.)	Bet:CA DES, 40% water	RT	24h	[98]
Freeze-dried grape pomace (*V. vinifera* L.)	ChCl:Oa DES, 30% water	60	10 min	[99]
Grape pomace (*V. vinifera* L.)	Bet:Glc DES, 30% water, UAE Bet:Scu DES 30% water, UAE	65	55 min	[100]
Artichoke heads (*C. scolymus* L.)	EtOH 80%	RT	1 h	[67]
Artichoke leaves and stalks (*C. scolymus* L.)	EtOH 50%	98	3 min	[71]
Freeze-dried artichoke bracts, leaves and stems (*C. scolymus* L.)	MeOH 60%, UAE	RT	30 min ultrasound pretreatment 1 h extraction	[61]
Oven-dried artichoke heads, leaves and stems (*C. scolymus* L.)	EtOH 25%, MAE	50	5 min	[63]
Olive tree leaves (*Olea europaea*)	30% acetonitrile, MAE	RT	1.5 min	[108]
Green tea plant (*Camellia sinensis*)	Ch-Cl/Gly DES, UAE	RT	21 min	[110]
Kiwi peel (*Actinidia deliciosa*)	34% EtOH, UAE	25	34.4 min	[111]
Rosemary leaves (*Rosmarinus officinalis* L.)	ChCl:1,2-propanediol DES, 50% water	65	150 min	[29]
Rosemary leaves (*Rosmarinus officinalis* L.)	refined soybean oil with 1% *w/w* of soy lecithin	40	3 h	[112]
Orange peel (*Citrus sinensis* L.)	ChCl:EG 1:4 DES, 10% water	60	100 min	[113]
Olive tree leaves (*Olea europaea*)	Gly/La 1:1 DES, 50% water, homogenizer-assisted extraction	RT	90 s	[109]
Onion solid wastes (*Allium cepa* L.)	GL/SP 8:1 DES	80	150 min	[114]
*Osmanthus fragrans* freeze-dried flowers	Glu/La 5:1 DES, 50% Ethanol, MAE	RT	15 s	[115]
Saffron crocus petals (*Crocus sativus)*	La/Gly 5:1 DES, 30% water	50	150 min	[116]

## Data Availability

Not applicable.
